# First-line pembrolizumab plus chemotherapy for advanced/metastatic esophageal cancer: 1-year extended follow-up in the Japanese subgroup of the phase 3 KEYNOTE-590 study

**DOI:** 10.1007/s10388-024-01053-z

**Published:** 2024-04-12

**Authors:** Ken Kato, Takashi Kojima, Hiroki Hara, Akihito Tsuji, Hisateru Yasui, Kei Muro, Taroh Satoh, Takashi Ogata, Ryu Ishihara, Masahiro Goto, Hideo Baba, Tomohiro Nishina, ShiRong Han, Keiichi Iwakami, Naoyoshi Yatsuzuka, Toshihiko Doi

**Affiliations:** 1 Department of Head and Neck, Esophageal Medical Oncology, National Cancer Center Hospital, 5-1-1, Tsukiji, Chuo-ku, Tokyo, 104-0045 Japan; 2 Department of Gastroenterology and Gastrointestinal Oncology, National Cancer Center Hospital East, Chiba, Japan; 3 Department of Gastroenterology, Saitama Cancer Center, Saitama, Japan; 4 Department of Medical Oncology, Kagawa University Hospital, Miki, Kagawa Japan; 5 Department of Medical Oncology, Kobe City Medical Center General Hospital, Kobe, Hyogo Japan; 6 Department of Clinical Oncology, Aichi Cancer Center Hospital, Nagoya, Aichi Japan; 7 Center for Cancer Genomics and Precision Medicine, Osaka University Hospital, Osaka, Japan; 8 Department of Gastrointestinal Surgery, Kanagawa Cancer Center, Yokohama, Kanagawa Japan; 9 Department of Gastrointestinal Oncology, Osaka International Cancer Institute, Osaka, Japan; 10 Cancer Chemotherapy Center, Osaka Medical and Pharmaceutical University Hospital, Osaka, Japan; 11 Department of Gastroenterological Surgery, Kumamoto University Hospital, Kumamoto, Japan; 12 Department of Gastrointestinal Medical Oncology, National Hospital Organization Shikoku Cancer Center, Matsuyama, Ehime Japan; 13 Department of Medical Oncology, MSD K.K., Tokyo, Japan

**Keywords:** Esophageal squamous cell carcinoma, Immune checkpoint inhibitors, Immunotherapy, Pembrolizumab, Programmed cell death ligand 1

## Abstract

**Background:**

First-line pembrolizumab plus chemotherapy (pembrolizumab–chemotherapy) demonstrated improved efficacy and a manageable safety profile versus placebo plus chemotherapy (placebo–chemotherapy) in the subgroup analysis of Japanese patients with advanced/metastatic esophageal cancer in KEYNOTE-590 at a median follow-up of 24.4 months. Longer-term data from the Japanese subgroup analysis of KEYNOTE-590 are reported.

**Methods:**

Patients were randomly assigned 1:1 to pembrolizumab 200 mg or placebo every 3 weeks for ≤ 35 cycles plus chemotherapy (cisplatin 80 mg/m^2^ and 5-fluorouracil 800 mg/m^2^/day). Endpoints included overall survival (OS) and progression-free survival (PFS; investigator-assessed per RECIST v1.1; dual primary) and safety (secondary). Early tumor shrinkage (ETS) and depth of response (DpR) were assessed post hoc.

**Results:**

Overall, 141 patients were enrolled in Japan. As of July 9, 2021, median follow-up was 36.6 months (range, 29.8–45.7). Pembrolizumab–chemotherapy showed a trend toward favorable OS (hazard ratio [HR], 0.70; 95% confidence interval [CI] 0.47–1.03) and PFS (0.57; 0.39–0.83) versus placebo–chemotherapy. In the pembrolizumab–chemotherapy group, patients with ETS ≥ 20% (55/74; 74.3%) versus < 20% (19/74; 25.7%) had favorable OS (HR, 0.23; 95% CI 0.12–0.42) and PFS (0.24; 0.13–0.43). Patients with DpR ≥ 60% (31/74; 41.9%) versus < 60% (43/74; 58.1%) had favorable OS (HR, 0.37; 95% CI 0.20–0.68) and PFS (0.24; 0.13–0.43). Grade 3–5 treatment-related adverse events occurred in 55/74 patients (74.3%) with pembrolizumab–chemotherapy and 41/67 patients (61.2%) with placebo–chemotherapy.

**Conclusions:**

With longer-term follow-up of Japanese patients with advanced/metastatic esophageal cancer, efficacy continued to favor pembrolizumab–chemotherapy compared with placebo–chemotherapy, with no new safety signals observed.

Clinical trial registration: ClinicalTrials.gov, NCT03189719.

**Supplementary Information:**

The online version contains supplementary material available at 10.1007/s10388-024-01053-z.

## Introduction

The programmed cell death protein 1 (PD-1) inhibitor pembrolizumab in combination with chemotherapy (pembrolizumab–chemotherapy) is currently a standard-of-care first-line treatment for patients with radically unresectable, advanced, or recurrent esophageal cancer based on the results of the randomized, placebo-controlled, phase 3 KEYNOTE-590 study [1–4]. With a median follow-up of 22.6 months, pembrolizumab–chemotherapy demonstrated statistically significant improvements in overall survival (OS), progression-free survival (PFS), and objective response rate (ORR) compared with placebo plus chemotherapy (placebo–chemotherapy), and had a manageable safety profile in patients with previously untreated advanced esophageal cancer [1]. Pembrolizumab–chemotherapy continued to provide efficacy benefit and a manageable safety profile compared with placebo–chemotherapy, with a median follow-up of 34.8 months [5].

Consistent with data in the global KEYNOTE-590 population [1], OS, PFS, and ORR favored pembrolizumab–chemotherapy compared with placebo–chemotherapy, and safety was manageable in the Japanese patient subgroup at a median follow-up of 24.4 months [6]. Median OS was 17.6 months in the pembrolizumab–chemotherapy group versus 11.7 months in the placebo–chemotherapy group in all randomly assigned patients (hazard ratio [HR], 0.71; 95% confidence interval [CI] 0.47–1.09), and median PFS was 6.3 months versus 6.0 months (0.58; 0.40–0.84); ORR was 56.8% versus 38.8% (between-group difference, 18.0%). The benefit of pembrolizumab–chemotherapy was also consistent in patients with esophageal squamous cell carcinoma (ESCC), programmed cell death ligand 1 (PD-L1) combined positive score (CPS) ≥ 10, and ESCC PD-L1 CPS ≥ 10 [6].

Early and/or continuous on-treatment measures that are predictive of longer-term efficacy outcomes in patients with advanced esophageal cancer are currently lacking. Such predictive measures may serve as valuable on-treatment endpoints. A trend toward an association with OS and PFS has been observed with early tumor shrinkage (ETS) and depth of response (DpR) with first-line treatment in metastatic colorectal cancer [7, 8], advanced gastric cancer [9], and more recently, with first-line chemotherapy in metastatic esophageal cancer [10]. The clinical utility of ETS and DpR with pembrolizumab–chemotherapy in patients with advanced/metastatic esophageal cancer is unclear.

Here, we report longer-term data from the subgroup analysis of Japanese patients in the KEYNOTE-590 study, with a median follow-up of 36.6 months. We also describe post hoc exploratory analyses of the association of ETS and DpR with efficacy outcomes for pembrolizumab–chemotherapy.

## Methods

### Study design, treatment, and patients

This is a subgroup analysis of Japanese patients in the KEYNOTE-590 study. The study design has been previously published [1, 6]. Briefly, eligible patients had treatment-naive, histologically or cytologically confirmed, locally advanced unresectable or metastatic esophageal adenocarcinoma, ESCC, or advanced or metastatic Siewert type I adenocarcinoma of the esophagogastric junction; measurable disease per Response Evaluation Criteria in Solid Tumors version 1.1 (RECIST v1.1) by investigator assessment; and Eastern Cooperative Oncology Group (ECOG) performance status of 0 or 1.

Patients were randomly assigned 1:1 to receive intravenous pembrolizumab 200 mg or placebo (saline) every 3 weeks (Q3W) for ≤ 35 cycles (~ 2 years) with chemotherapy (cisplatin 80 mg/m^2^ intravenously Q3W for ≤ 6 doses and 5-fluorouracil 800 mg/m^2^/day continuous intravenous infusion on days 1–5 Q3W per local standard of care) until disease progression, unacceptable toxicity, or withdrawal of consent. Randomization was stratified by geographic region (Asia vs non-Asia), histology (adenocarcinoma vs ESCC), and ECOG performance status (0 vs 1).

### Outcomes and assessments

The dual primary endpoints were OS and PFS per RECIST v1.1 by investigator assessment in all randomly assigned patients, patients with ESCC, and patients with PD-L1 CPS ≥ 10, and OS in patients with ESCC PD-L1 CPS ≥ 10. Secondary endpoints included ORR and duration of response (DOR) per RECIST v1.1 by investigator assessment in all randomly assigned patients and patients with ESCC, PD-L1 CPS ≥ 10, and ESCC PD-L1 CPS ≥ 10, and safety and tolerability in all treated patients. Assessment details of these endpoints have been previously described [1, 11]. Post hoc exploratory analyses included the association of efficacy outcomes with ETS (defined as percentage decrease in the sum of the target lesions’ longest diameter after 9 weeks [± 7 days] from randomization date—the protocol-specified timepoint for the first on-study imaging assessment) and DpR (defined as percentage of the maximal tumor shrinkage during the trial) in patients assigned to receive pembrolizumab–chemotherapy. PD-L1 expression was assessed in archival or newly collected tumor samples using PD-L1 IHC 22C3 pharmDx (Agilent) and measured using CPS (defined as the number of PD-L1–staining cells [tumor cells, lymphocytes, macrophages] divided by the total number of viable tumor cells, multiplied by 100).

### Statistical analysis

Efficacy was evaluated in the intention-to-treat population. Safety was assessed in all randomly assigned patients who received ≥ 1 dose of study treatment. Overall survival and PFS were estimated using the nonparametric Kaplan–Meier method; the magnitude of the treatment difference (HR) was determined using a Cox proportional hazards model with the Efron method for handling ties. Subgroup analysis was not controlled for multiplicity; no alpha was allocated to the comparisons. Details of the sample size calculation are provided in Online Resource 1.

Data cutoff for this analysis was July 9, 2021. This trial is registered with ClinicalTrials.gov (NCT03189719).

## Results

### Patients

Of 749 patients enrolled globally in KEYNOTE-590 [1], 141 were enrolled in Japan (pembrolizumab–chemotherapy, 74 [52.5%]; placebo–chemotherapy, 67 [47.5%]). Baseline characteristics were generally balanced between treatment groups and have been previously reported [6]. Of the 74 patients assigned to the pembrolizumab–chemotherapy group, 67 (90.5%) had ESCC, 48 (64.9%) had PD-L1 CPS ≥ 10, and 44 (59.5%) had ESCC PD-L1 CPS ≥ 10. Of the 67 patients assigned to the placebo–chemotherapy group, 59 (88.1%) had ESCC, 36 (53.7%) had PD-L1 CPS ≥ 10, and 32 (47.8%) had ESCC PD-L1 CPS ≥ 10.

Median follow-up, defined as the time from randomization to the data cutoff date, was 36.6 months (range, 29.8–45.7). At the data cutoff date, 64 of 74 patients (86.5%) in the pembrolizumab–chemotherapy group and 67 patients (100%) in the placebo–chemotherapy group had discontinued treatment, most commonly due to disease progression (pembrolizumab–chemotherapy, 43/74 [58.1%]; placebo–chemotherapy, 54/67 [80.6%]) (Online Resource 2). Nine of 74 patients (12.2%) in the pembrolizumab–chemotherapy group and no patients in the placebo–chemotherapy group completed 35 cycles of treatment; treatment was ongoing for 1 of 74 patients (1.4%) and no patients, respectively (Online Resource 2).

Among patients who discontinued treatment, 47 of 64 patients (73.4%) in the pembrolizumab–chemotherapy group and 52 of 67 patients (77.6%) in the placebo–chemotherapy group received subsequent anticancer therapy (Online Resource 3); paclitaxel was the most common subsequent anticancer drug received and nivolumab was the most common subsequent immunotherapy received.

### Overall survival

**Fig. 1:**
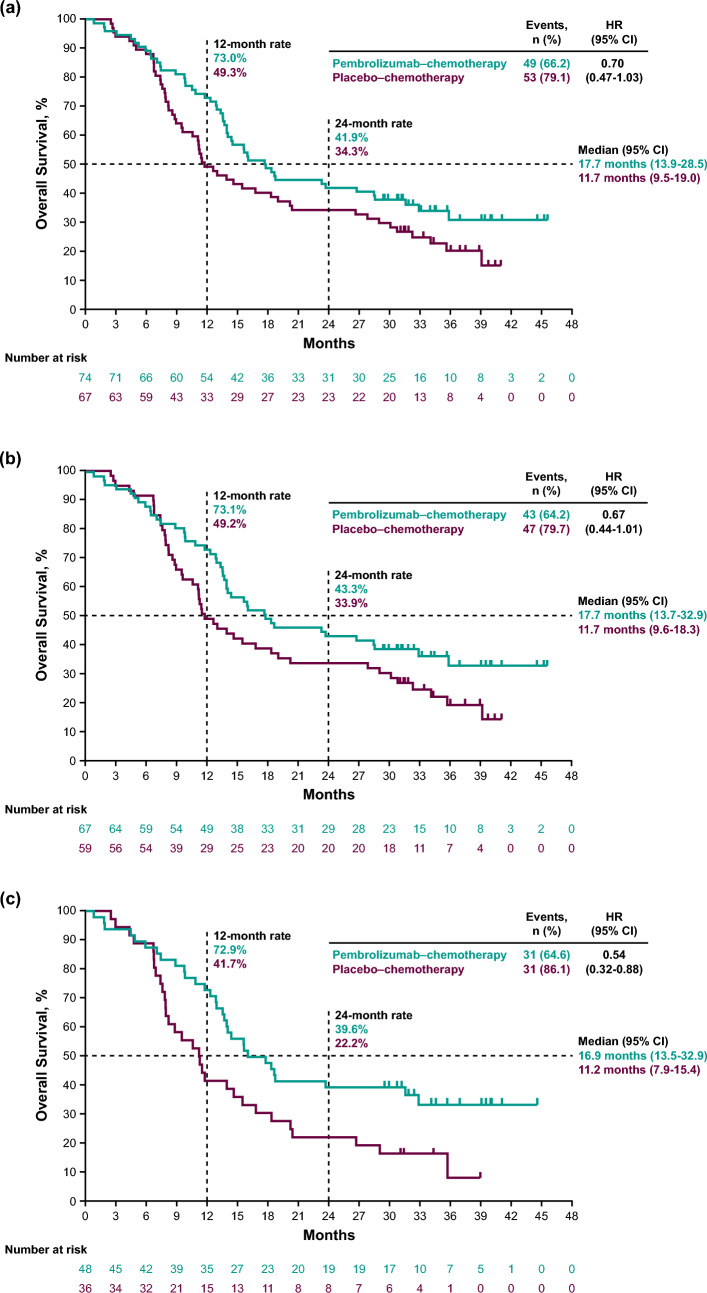

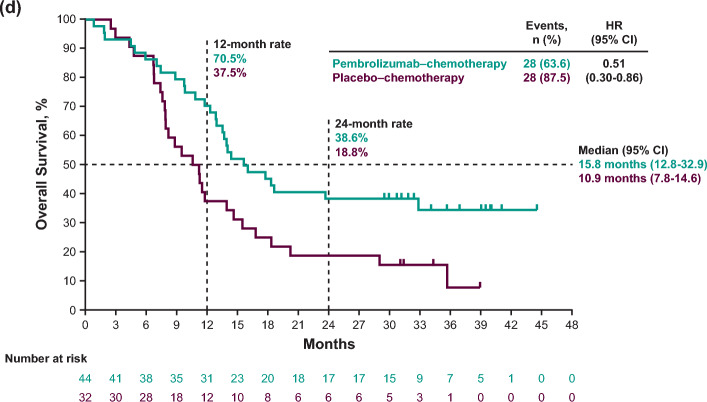
Kaplan–Meier estimates of overall survival. **a** All randomly assigned patients. **b** Patients with ESCC. **c** Patients with PD-L1 CPS ≥ 10. **d** Patients with ESCC PD-L1 CPS ≥ 10. Events were defined as patients who died. *CI* confidence interval, *CPS* combined positive score, *ESCC* esophageal squamous cell carcinoma, *HR* hazard ratio, *PD-L1* programmed cell death ligand 1, *pembrolizumab–chemotherapy* pembrolizumab plus chemotherapy, *placebo–chemotherapy* placebo plus chemotherapy

Median OS in the pembrolizumab–chemotherapy group was 17.7 months (95% CI 13.9–28.5) versus 11.7 months (9.5–19.0) in the placebo–chemotherapy group in all randomly assigned patients (HR, 0.70; 95% CI 0.47–1.03) (Fig. 1a), 17.7 months (13.7–32.9) versus 11.7 months (9.6–18.3) in patients with ESCC (0.67; 0.44–1.01) (Fig. 1b), 16.9 months (13.5–32.9) versus 11.2 months (7.9–15.4) in patients with PD-L1 CPS ≥ 10 (0.54; 0.32–0.88) (Fig. 1c), and 15.8 months (12.8–32.9) versus 10.9 months (7.8–14.6) in patients with ESCC PD-L1 CPS ≥ 10 (0.51; 0.30–0.86) (Fig. 1d). The estimated 24-month OS rate in the pembrolizumab–chemotherapy group versus placebo–chemotherapy group was 41.9% versus 34.3% in all randomly assigned patients, 43.3% versus 33.9% in patients with ESCC, 39.6% versus 22.2% in patients with PD-L1 CPS ≥ 10, and 38.6% versus 18.8% in patients with ESCC PD-L1 CPS ≥ 10.

### Progression-free survival

**Fig. 2:**
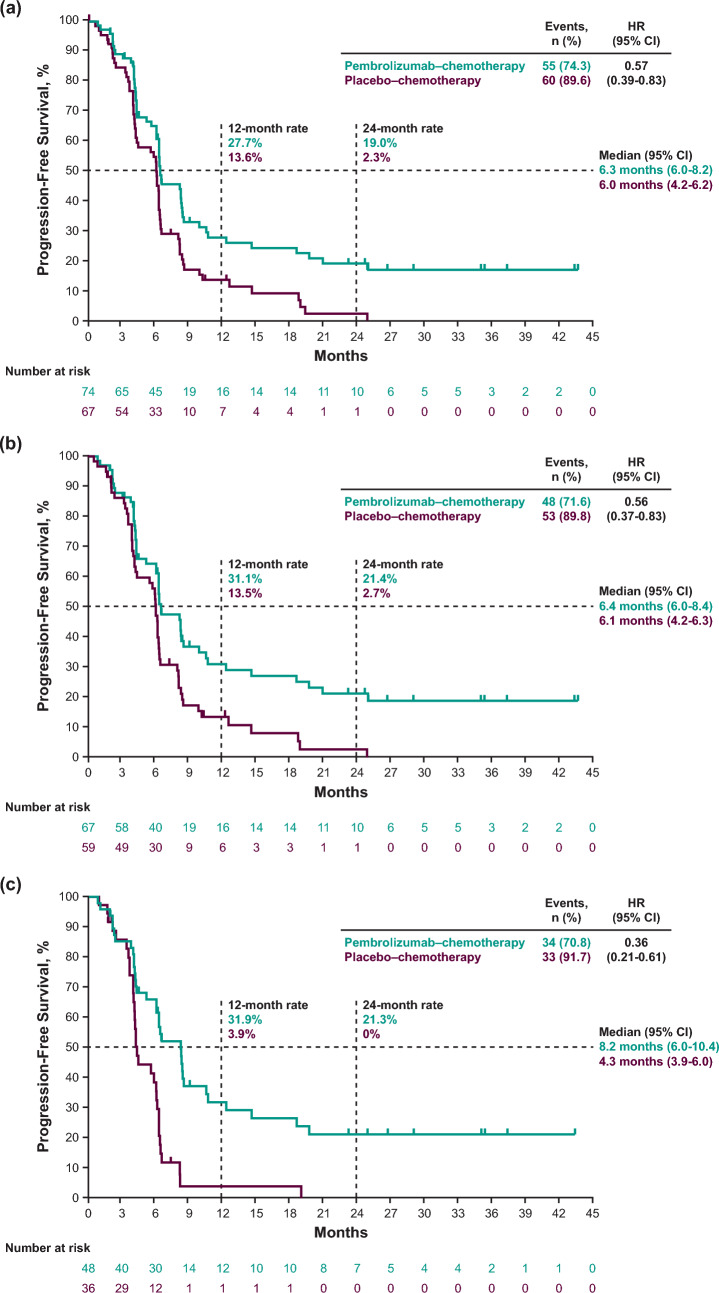
Kaplan–Meier estimates of progression-free survival. **a** All randomly assigned patients. **b** Patients with ESCC. **c** Patients with PD-L1 CPS ≥ 10. Events were defined as patients with progressive disease or patients who died. *CI* confidence interval, *CPS* combined positive score, *ESCC* esophageal squamous cell carcinoma, *HR* hazard ratio, *PD-L1* programmed cell death ligand 1, *pembrolizumab–chemotherapy* pembrolizumab plus chemotherapy, *placebo–chemotherapy* placebo plus chemotherapy

Median PFS in the pembrolizumab–chemotherapy group was 6.3 months (95% CI 6.0–8.2) versus 6.0 months (4.2–6.2) in the placebo–chemotherapy group in all randomly assigned patients (HR, 0.57; 95% CI 0.39–0.83) (Fig. 2a), 6.4 months (6.0–8.4) versus 6.1 months (4.2–6.3) in patients with ESCC (0.56; 0.37–0.83) (Fig. 2b), and 8.2 months (6.0–10.4) versus 4.3 months (3.9–6.0) in patients with PD-L1 CPS ≥ 10 (0.36; 0.21–0.61) (Fig. 2c). The estimated 24-month PFS rate in the pembrolizumab–chemotherapy group versus placebo–chemotherapy group was 19.0% versus 2.3% in all randomly assigned patients, 21.4% versus 2.7% in patients with ESCC, and 21.3% versus 0% in patients with PD-L1 CPS ≥ 10.

### Response

Among all randomly assigned patients, ORR was 56.8% (42/74; 4 complete responses [CRs]; 38 partial responses [PRs]) in the pembrolizumab–chemotherapy group and 38.8% (26/67; 3 CRs; 23 PRs) in the placebo–chemotherapy group; median DOR (range) was 8.3 months (1.2 + to 41.7 +) and 6.1 months (3.5 to 22.8), respectively. In patients with ESCC, ORR was 56.7% (38/67; 4 CRs; 34 PRs) in the pembrolizumab–chemotherapy group and 40.7% (24/59; 2 CRs; 22 PRs) in the placebo–chemotherapy group; median DOR (range) was 10.4 months (1.2 + to 41.7 +) and 6.1 months (3.5 to 22.8), respectively. In patients with PD-L1 CPS ≥ 10, ORR was 60.4% (29/48; 3 CRs; 26 PRs) in the pembrolizumab–chemotherapy group and 30.6% (11/36; 1 CR; 10 PRs) in the placebo–chemotherapy group; median DOR (range) was 10.4 months (2.3 + to 41.3 +) and 4.4 months (3.5 to 17.0), respectively. In patients with ESCC PD-L1 CPS ≥ 10, ORR was 59.1% (26/44; 3 CRs; 23 PRs) in the pembrolizumab–chemotherapy group and 31.3% (10/32; 1 CR; 9 PRs) in the placebo–chemotherapy group; median DOR (range) was 10.5 months (2.3 + to 41.3 +) and 4.4 months (3.5 to 17.0), respectively.

### Efficacy by ETS and DpR in the pembrolizumab–chemotherapy group

**Fig. 3:**
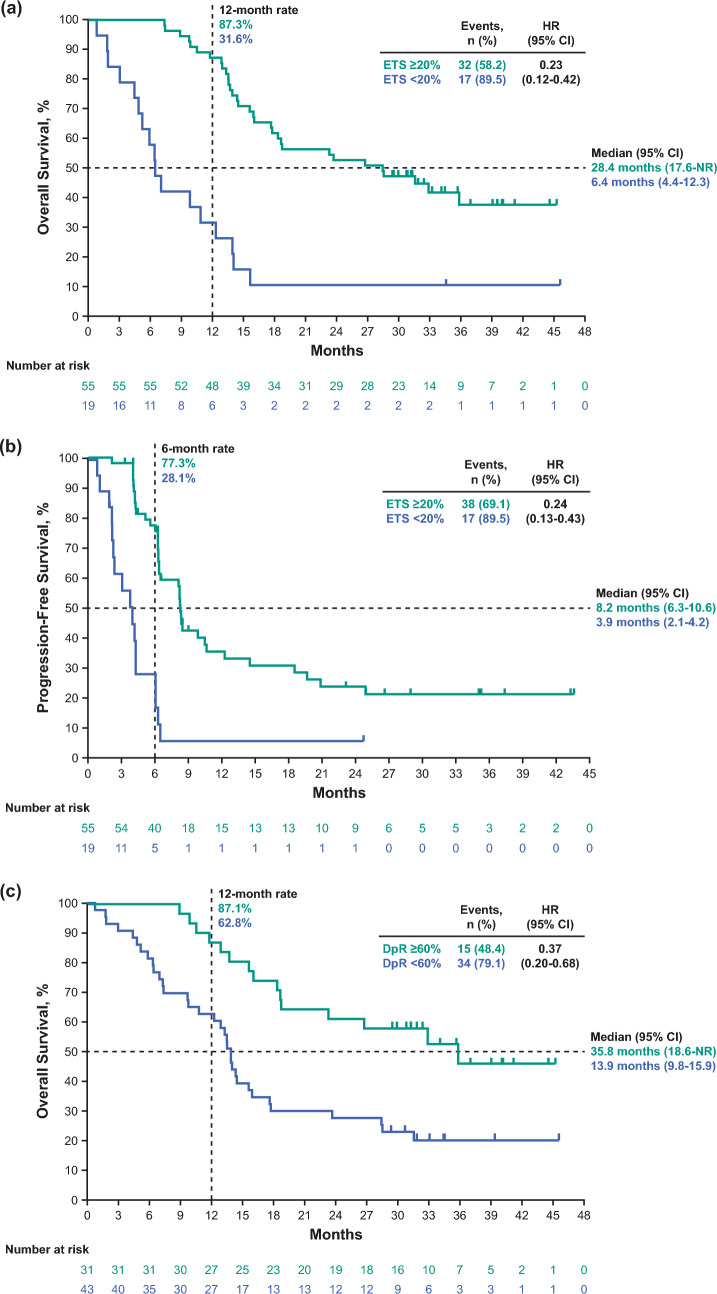

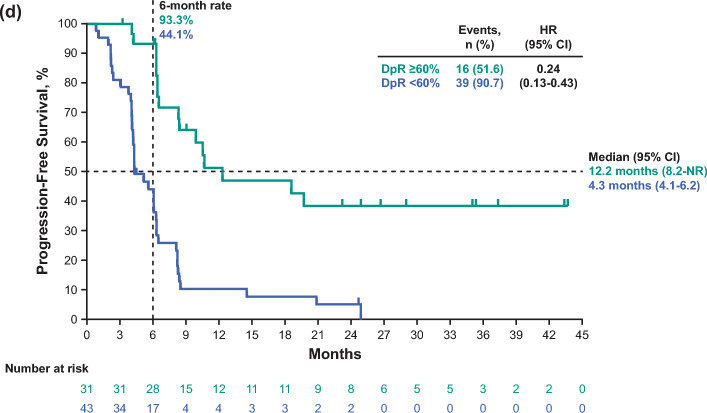
Kaplan–Meier estimates of survival in all randomly assigned patients in the pembrolizumab*–*chemotherapy group by ETS cutoff of 20% and DpR cutoff of 60%. **a** OS by ETS ≥ 20% versus < 20%. **b** PFS by ETS ≥ 20% versus < 20%. **c** OS by DpR ≥ 60% versus < 60%. **d** PFS by DpR ≥ 60% versus < 60%. For OS, events were defined as patients who died. For PFS, events were defined as patients with progressive disease or patients who died. *CI* confidence interval, *DpR* depth of response, *ETS* early tumor shrinkage, *HR* hazard ratio, *NR* not reached, *OS* overall survival*, PD-L1* programmed cell death ligand 1, *pembrolizumab–chemotherapy* pembrolizumab plus chemotherapy, *PFS* progression-free survival

Median OS and PFS by ETS cutoffs in the pembrolizumab–chemotherapy group are presented in Online Resource 4. The cutoff value of 20% for ETS was chosen because, in this study, the hazard ratios of PFS and OS at this cutoff were the smallest. Of 74 patients in the pembrolizumab–chemotherapy group, 55 (74.3%) had an ETS ≥ 20% and 19 (25.7%) had an ETS < 20%. When comparing ETS ≥ 20% versus < 20%, median OS (95% CI) was 28.4 months (17.6-not reached [NR]) versus 6.4 months (4.4–12.3; HR, 0.23; 95% CI, 0.12–0.42) (Fig. 3a); median PFS (95% CI) was 8.2 months (6.3–10.6) versus 3.9 months (2.1–4.2; HR, 0.24; 95% CI, 0.13–0.43) (Fig. 3b).

Median OS and PFS by DpR cutoffs in the pembrolizumab–chemotherapy group are presented in Online Resource 5. The cutoff of 60% was chosen because the values above and below this cutoff were well-balanced, and, in this study, the hazard ratios of PFS and OS at DpR 60% were smaller. Of 74 patients in the pembrolizumab–chemotherapy group, 31 (41.9%) had a DpR ≥ 60% and 43 (58.1%) had a DpR < 60%. When comparing DpR ≥ 60% versus < 60%, median OS (95% CI) was 35.8 months (18.6-NR) versus 13.9 months (9.8–15.9; HR, 0.37; 95% CI, 0.20–0.68) (Fig. 3c); median PFS (95% CI) was 12.2 months (8.2-NR) versus 4.3 months (4.1–6.2; HR, 0.24; 95% CI 0.13–0.43) (Fig. 3d). Comparisons of response by ETS and DpR cutoffs are presented in Online Resource 6.

**Fig. 4:**
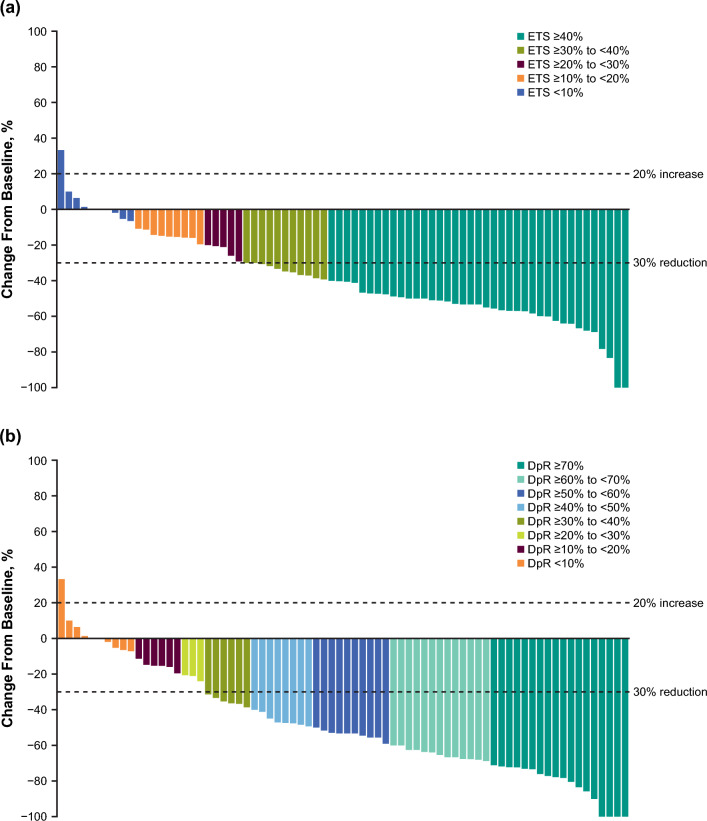
Change from baseline in tumor measurements per RECIST v1.1 by investigator-assessment in the pembrolizumab*–*chemotherapy group. **a** Percentage change at 9 weeks by ETS cutoffs. **b** Maximum percentage change during treatment course by DpR cutoffs. For ETS assessment of 3 patients who had only 1 imaging assessment before 63 days–7 days from the randomization date, the first imaging assessment was used. For ETS assessment of 1 patient who had 2 imaging assessments after 63 days ± 7 days from the randomization date, the earlier imaging assessment was used; both percentage decreases were less than 10%. *DpR* depth of response, *ETS* early tumor shrinkage, *pembrolizumab–chemotherapy* pembrolizumab plus chemotherapy, *RECIST v1*.*1* Response Evaluation Criteria in Solid Tumors version 1.1

In the pembrolizumab–chemotherapy group, 50 of 74 patients (67.6%) had a ≥ 30% reduction from baseline in the sum of target lesions’ longest diameter at 9 weeks (Fig. 4a); 55 of 74 patients (74.3%) had a ≥ 30% maximum reduction from baseline in target lesion size during the trial (Fig. 4b). The longitudinal percentage change from baseline in target lesion is presented in Online Resource 7.

### Safety

Adverse events (AEs) occurred in all patients in the pembrolizumab–chemotherapy and placebo–chemotherapy groups (Online Resource 8). Treatment-related AEs occurred in 73 of 74 patients (98.6%) in the pembrolizumab–chemotherapy group and 66 of 67 patients (98.5%) in the placebo–chemotherapy group. Grade 3–5 treatment-related AEs occurred in 55 of 74 patients (74.3%) in the pembrolizumab–chemotherapy group and 41 of 67 patients (61.2%) in the placebo–chemotherapy group; grade 5 treatment-related AEs occurred in 2 of 74 patients (2.7%; interstitial lung disease and pneumonitis) and 1 of 67 patients (1.5%; interstitial lung disease), respectively (Table 1). Treatment-related AEs led to discontinuation in 16 of 74 patients (21.6%) in the pembrolizumab–chemotherapy group and 12 of 67 patients (17.9%) in the placebo–chemotherapy group. The most common treatment-related AEs in the pembrolizumab–chemotherapy and placebo–chemotherapy groups were decreased appetite (78.4% and 58.2%, respectively) and nausea (74.3% and 62.7%) (Table 1).

**Table 1:** Adverse events in Japanese patients

Events, n (%)	Pembrolizumab–chemotherapy, *n* = 74		Placebo–chemotherapy, *n* = 67	
	Any grade	Grade ≥ 3	Any grade	Grade ≥ 3
Treatment-related AEs^a^	73 (98.6)	55 (74.3)	66 (98.5)	41 (61.2)
Decreased appetite	58 (78.4)	4 (5.4)	39 (58.2)	7 (10.4)
Nausea	55 (74.3)	2 (2.7)	42 (62.7)	3 (4.5)
Decreased neutrophil count	45 (60.8)	29 (39.2)	38 (56.7)	22 (32.8)
Stomatitis	42 (56.8)	3 (4.1)	35 (52.2)	2 (3.0)
Decreased WBC count	35 (47.3)	14 (18.9)	22 (32.8)	6 (9.0)
Anemia	28 (37.8)	11 (14.9)	29 (43.3)	11 (16.4)
Fatigue	26 (35.1)	2 (2.7)	10 (14.9)	1 (1.5)
Malaise	26 (35.1)	1 (1.4)	23 (34.3)	2 (3.0)
Constipation	23 (31.1)	0	19 (28.4)	0
Hiccups	23 (31.1)	0	15 (22.4)	0
Diarrhea	20 (27.0)	3 (4.1)	18 (26.9)	2 (3.0)
Increased blood creatinine	20 (27.0)	0	22 (32.8)	0
Alopecia	18 (24.3)	0	13 (19.4)	0
Decreased platelet count	17 (23.0)	1 (1.4)	14 (20.9)	1 (1.5)
Dysgeusia	16 (21.6)	0	13 (19.4)	0
Peripheral sensory neuropathy	15 (20.3)	1 (1.4)	14 (20.9)	0
Decreased lymphocyte count	12 (16.2)	6 (8.1)	8 (11.9)	2 (3.0)
Edema	11 (14.9)	0	8 (11.9)	0
Hyponatremia	10 (13.5)	9 (12.2)	16 (23.9)	8 (11.9)
Vomiting	8 (10.8)	1 (1.4)	10 (14.9)	0
Vasculitis	6 (8.1)	0	7 (10.4)	0
Rash	5 (6.8)	0	8 (11.9)	0
Hyperkalemia	1 (1.4)	1 (1.4)	7 (10.4)	2 (3.0)
Immune-mediated AEs and infusion reactions^b^	25 (33.8)	10 (13.5)	17 (25.4)	1 (1.5)
Hypothyroidism	7 (9.5)	0	5 (7.5)	0
Vasculitis	6 (8.1)	0	8 (11.9)	0
Pneumonitis	5 (6.8)	3 (4.1)	1 (1.5)	1 (1.5)
Colitis	4 (5.4)	2 (2.7)	1 (1.5)	0
Severe skin reactions	4 (5.4)	4 (5.4)	0	0
Hyperthyroidism	3 (4.1)	0	1 (1.5)	0
Hypophysitis	2 (2.7)	0	0	0
Infusion reactions	2 (2.7)	0	1 (1.5)	0
Adrenal insufficiency	1 (1.4)	1 (1.4)	2 (3.0)	0
Hepatitis	1 (1.4)	1 (1.4)	0	0
Type 1 diabetes mellitus	1 (1.4)	1 (1.4)	0	0
Nephritis	0	0	1 (1.5)	0

Two of 74 patients (2.7%) in the pembrolizumab–chemotherapy group (interstitial lung disease and pneumonitis, n = 1 each) and 1 of 67 patients (1.5%) in the placebo–chemotherapy group (interstitial lung disease, n = 1) died due to a treatment-related AE. Two of 74 patients (2.7%) in the pembrolizumab–chemotherapy group (both due to pneumonitis) and 1 of 67 patients (1.5%) in the placebo–chemotherapy group (due to pneumonitis) died due to immune-mediated AEs and infusion reactions

*AE* adverse event, *pembrolizumab–chemotherapy* pembrolizumab plus chemotherapy, *placebo–chemotherapy* placebo plus chemotherapy, *WBC* white blood cell

^a^Determined by the investigator to be related to the drug. Treatment-related AEs with an incidence of ≥ 10% in either treatment group are shown

^b^Immune-mediated AEs and infusion reactions were based on a list of preferred terms intended to capture known risks of pembrolizumab and were considered regardless of attribution to any study treatment by the investigator

Immune-mediated AEs and infusion reactions occurred in 25 of 74 patients (33.8%) in the pembrolizumab–chemotherapy group and 17 of 67 patients (25.4%) in the placebo–chemotherapy group. Grade 3–5 immune-mediated AEs and infusion reactions occurred in 10 of 74 patients (13.5%) in the pembrolizumab–chemotherapy group and 1 of 67 patients (1.5%) in the placebo–chemotherapy group; grade 5 immune-mediated AEs and infusion reactions occurred in 2 of 74 patients (2.7%; both pneumonitis) and 1 of 67 patients (1.5%; pneumonitis), respectively (Table 1). Immune-mediated AEs and infusion reactions led to discontinuation in 7 of 67 patients (9.5%) in the pembrolizumab–chemotherapy group and 2 of 67 patients (3.0%) in the placebo–chemotherapy group. The most common immune-mediated AEs and infusion reactions in the pembrolizumab–chemotherapy and placebo–chemotherapy groups were hypothyroidism (9.5% and 7.5%, respectively) and vasculitis (8.1% and 11.9%) (Table 1).

## Discussion

With a median follow-up of 36.6 months in the Japan subgroup in the KEYNOTE-590 study of patients with advanced/metastatic esophageal cancer, treatment with pembrolizumab–chemotherapy prolonged OS and PFS and provided durable responses compared to placebo–chemotherapy; no new safety signals were observed. Additionally, post hoc exploratory analyses in the pembrolizumab–chemotherapy group showed that a relatively high percentage of patients (74.3%) had an ETS ≥ 20%, and median OS and PFS trended numerically higher in patients with higher versus lower ETS or DpR cutoff values.

When compared to the previous analysis (median follow-up, 24.4 months) in patients enrolled in Japan [6], the HR estimates for OS and PFS in the pembrolizumab–chemotherapy group versus the placebo–chemotherapy group were similar in all randomly assigned patients (OS: 0.70 and 0.71; PFS: 0.57 and 0.58) and in patients with ESCC (OS: 0.67 and 0.69; PFS: 0.56 and 0.57), PD-L1 CPS ≥ 10 (OS: 0.54 and 0.58; PFS: 0.36 and 0.36), and ESCC PD-L1 CPS ≥ 10 (OS: 0.51 and 0.55; PFS: not assessed). Pembrolizumab–chemotherapy also continued to demonstrate a higher ORR compared with placebo–chemotherapy in all randomly assigned patients (56.8% vs 38.8%) and in patients with ESCC (56.7% vs 40.7%), PD-L1 CPS ≥ 10 (60.4% vs 30.6%), and ESCC PD-L1 CPS ≥ 10 (59.1% vs 31.3%). Additionally, pembrolizumab–chemotherapy continued to provide durable responses, with ≥ 26.7% of all randomly assigned patients and patients with ESCC, PD-L1 CPS ≥ 10, and ESCC PD-L1 CPS ≥ 10 continuing to respond for ≥ 24 months per Kaplan–Meier estimates.

Compared to placebo–chemotherapy, observed OS, PFS, and ORR improvements with pembrolizumab–chemotherapy were consistent with those of the global KEYNOTE-590 population [1, 5] and were comparable to results from other randomized studies for PD-1 inhibitors plus chemotherapy as first-line treatment of patients with advanced esophageal cancer from global and Japanese populations [12–17]. Comparisons between this study and published studies should be made with caution, given study differences such as patient characteristics, study design, and treatment regimen.

The relatively high percentage of patients with a higher ETS cutoff and the trend toward numerically longer median OS and PFS for ETS ≥ 20% versus < 20% and for DpR ≥ 60% versus < 60% suggest that ETS and DpR may be associated with improved OS and PFS with pembrolizumab–chemotherapy in patients with advanced/metastatic esophageal cancer. A similar trend of an association between greater ETS and DpR cutoffs and improved efficacy outcomes has been reported in patients with metastatic esophageal cancer treated with first-line chemotherapy [10] and in patients with other tumor types [7, 9, 18–21]. Data from the present study suggest that ETS and DpR may have an early and continuous on-treatment utility, respectively, as surrogate endpoints in patients with advanced/metastatic esophageal cancer treated with first-line pembrolizumab–chemotherapy; however, further studies are needed.

With longer-term follow-up in the Japan subgroup, the AE profile of pembrolizumab–chemotherapy remained consistent over time [6]. Additionally, the AE profile of pembrolizumab–chemotherapy in the Japan subgroup is similar to that of a longer-term analysis in the global population [5] and is consistent with the known AE profile of pembrolizumab–chemotherapy [22, 23].

This subgroup analysis is limited by the low number of patients enrolled in Japan. Although not prespecified, this is the first report of an ETS or DpR analysis in patients with esophageal cancer treated with a PD-1 inhibitor. Limitations of the post hoc exploratory analyses include the use of 9 weeks to define ETS (several other reports have used ≤ 8 weeks [7, 8, 10]) and the lack of a corresponding analysis in the placebo–chemotherapy group.

In conclusion, OS, PFS, and ORR continued to favor pembrolizumab–chemotherapy compared to placebo–chemotherapy, and safety remained consistent over time with longer-term follow-up of the Japan subgroup in the KEYNOTE-590 study of patients with advanced/metastatic esophageal cancer. The benefit of pembrolizumab–chemotherapy in the Japan subgroup is consistent with data in the global KEYNOTE-590 population. Additionally, ETS ≥ 20% at week 9 and trends in ETS and DpR are suggestive of an association with improved OS and PFS for pembrolizumab–chemotherapy in patients with advanced/metastatic esophageal cancer in the Japan subgroup. Taken together, the results reinforce the favorable benefit–risk profile of first-line pembrolizumab–chemotherapy in patients with unresectable advanced/metastatic esophageal cancer.

### Supplementary Information

Below is the link to the electronic supplementary material.

Supplementary file1 (PDF 728 KB)

## Data Availability

Merck Sharp & Dohme LLC, a subsidiary of Merck & Co., Inc., Rahway, NJ, USA (MSD), is committed to providing qualified scientific researchers access to anonymized data and clinical study reports from the company’s clinical trials for the purpose of conducting legitimate scientific research. MSD is also obligated to protect the rights and privacy of trial participants and, as such, has a procedure in place for evaluating and fulfilling requests for sharing company clinical trial data with qualified external scientific researchers. The MSD data sharing website (available at: http://engagezone.msd.com/ds_documentation.php) outlines the process and requirements for submitting a data request. Applications will be promptly assessed for completeness and policy compliance. Feasible requests will be reviewed by a committee of MSD subject matter experts to assess the scientific validity of the request and the qualifications of the requestors. In line with data privacy legislation, submitters of approved requests must enter into a standard data-sharing agreement with MSD before data access is granted. Data will be made available for request after product approval in the United States and the European Union or after product development is discontinued. There are circumstances that may prevent MSD from sharing requested data, including country or region-specific regulations. If the request is declined, it will be communicated to the investigator. Access to genetic or exploratory biomarker data requires a detailed, hypothesis-driven statistical analysis plan that is collaboratively developed by the requestor and MSD subject matter experts; after approval of the statistical analysis plan and execution of a data-sharing agreement, MSD will either perform the proposed analyses and share the results with the requestor or will construct biomarker covariates and add them to a file with clinical data that is uploaded to an analysis portal so that the requestor can perform the proposed analyses.
