# Supplementary material for: First-line pembrolizumab plus chemotherapy for advanced/metastatic esophageal cancer: 1-year extended follow-up in the Japanese subgroup of the phase 3 KEYNOTE-590 study
Source: Esophagus. 2024 Apr 12;21(3):306–18. doi: 10.1007/s10388-024-01053-z (PMC11199245; doi:10.1007/s10388-024-01053-z)
Supplement: Supplementary file 1 — Supplementary file1 (PDF 728 KB) [file 10388_2024_1053_MOESM1_ESM.pdf]

Online supplemental material

Journal: *Esophagus*

First-line pembrolizumab plus chemotherapy for advanced/metastatic esophageal cancer: 1-year extended follow-up in the Japanese subgroup of the phase 3 KEYNOTE-590 study

Ken Kato[1], Takashi Kojima[2], Hiroki Hara[3], Akihito Tsuji[4], Hisateru Yasui[5], Kei Muro[6], Taroh Satoh[7], Takashi Ogata[8], Ryu Ishihara[9], Masahiro Goto[10], Hideo Baba[11], Tomohiro Nishina[12], ShiRong Han[13], Keiichi Iwakami[13], Naoyoshi Yatsuzuka[13], Toshihiko Doi[2]

[1]Department of Head and Neck, Esophageal Medical Oncology, National Cancer Center Hospital, Tokyo, Japan; [2]Department of Gastroenterology and Gastrointestinal Oncology, National Cancer Center Hospital East, Chiba, Japan; [3]Department of Gastroenterology, Saitama Cancer Center, Saitama, Japan; [4]Department of Medical Oncology, Kagawa University Hospital, Kagawa, Japan; [5]Department of Medical Oncology, Kobe City Medical Center General Hospital, Hyogo, Japan; [6]Department of Clinical Oncology, Aichi Cancer Center Hospital, Aichi, Japan; [7]Center for Cancer Genomics and Precision Medicine, Osaka University Hospital, Osaka, Japan; [8]Department of Gastrointestinal Surgery, Kanagawa Cancer Center, Kanagawa, Japan; [9]Department of Gastrointestinal Oncology, Osaka International Cancer Institute, Osaka, Japan; [10]Cancer Chemotherapy Center, Osaka Medical and Pharmaceutical University Hospital, Osaka, Japan; [11]Department of Gastroenterological Surgery, Kumamoto University Hospital, Kumamoto, Japan; [12]Department of Gastrointestinal Medical Oncology, National Hospital Organization Shikoku Cancer Center, Ehime, Japan; [13]Department of Medical Oncology, MSD K.K., Tokyo, Japan

Corresponding Author: Ken Kato, MD, PhD

Department of Head and Neck, Esophageal Medical Oncology

National Cancer Center Hospital

5-1-1, Tsukiji, Chuo-ku

Tokyo, 104-0045, Japan

Phone: 03-3542-2511 (7902)

Fax: 03-3452-3815

Email: kenkato@ncc.go.jp

Online Resource 1 Sample size calculation for the Japan subgroup analysis

The estimated sample size of the Japan subgroup population was calculated to guarantee a >80% probability of consistency between the overall population and the Japan subgroup population on the primary endpoint of overall survival. Consistency was defined as the estimated hazard ratios for the overall population and the Japan subgroup population are both <1.

The necessary sample sizes to achieve >80% probability of consistency in the subgroup of patients with esophageal squamous cell carcinoma (ESCC) programmed cell death ligand 1 (PD-L1) combined positive score (CPS) ≥10 and patients with ESCC were 55 and 45, respectively. With 141 patients from Japan enrolled into the study, the probabilities increased to 91.6% for patients with ESCC PD-L1 CPS ≥10 and 93.6% for patients with ESCC.

Online Resource 2 CONSORT diagram

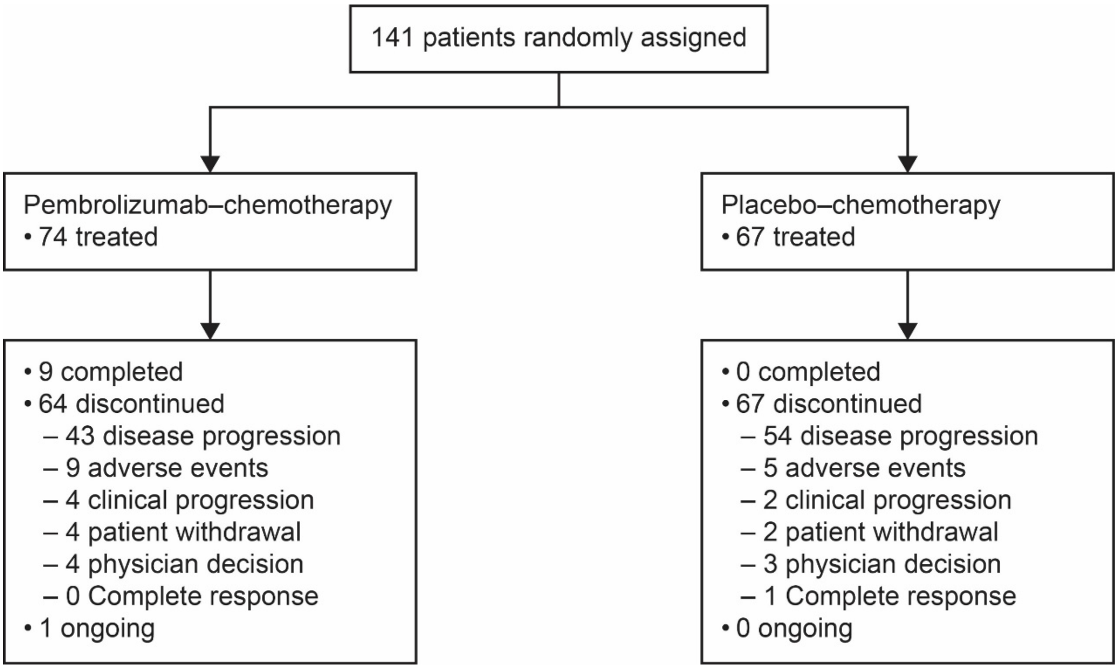

Pembrolizumab–chemotherapy pembrolizumab plus chemotherapy, *placebo–chemotherapy* placebo plus chemotherapy

Complete response is based on confirmed investigator timepoint assessment per Response Evaluation Criteria in Solid Tumors version 1.1.

Online Resource 3 Subsequent anticancer therapy in Japanese patients who discontinued study treatment

**Therapy, n (%)**	**Pembrolizumab–chemotherapy** ***n*** **= 64[a]**	**Placebo–chemotherapy** ***n*** **= 67[a]**
**All patients**	47 (73.4)	52 (77.6)
**Incidence ≥5% in any treatment group**		
Paclitaxel	35 (54.7)	39 (58.2)
Fluorouracil	21 (32.8)	15 (22.4)
Cisplatin	11 (17.2)	13 (19.4)
Nivolumab	11 (17.2)	16 (23.9)
Oxaliplatin	10 (15.6)	2 (3.0)
Levoleucovorin calcium	9 (14.1)	2 (3.0)
Docetaxel	5 (7.8)	8 (11.9)
Gimeracil (+) oteracil potassium (+) tegafur	5 (7.8)	6 (9.0)
Ramucirumab	5 (7.8)	5 (7.5)
**Immunotherapy incidence ≥0%**		
Nivolumab	11 (17.2)	16 (23.9)
Pembrolizumab	2 (3.1)	0
Ezabenlimab	0	1 (1.5)
Miptenalimab	0	1 (1.5)

Pembrolizumab–chemotherapy pembrolizumab plus chemotherapy, *placebo–chemotherapy* placebo plus chemotherapy

[a]64 of 74 patients (86.5%) in the pembrolizumab–chemotherapy group and 67 patients (100%) in the placebo–chemotherapy group discontinued treatment.

Online Resource 4 OS and PFS by ETS cutoffs in all randomized patients in the pembrolizumab–chemotherapy group

Categories	Patients, n	Median, months	HR (95% CI)
**OS**			
≥10% vs <10%	64 vs 10	21.0 vs 6.4	0.28 (0.13-0.59)
≥20% vs <20%	55 vs 19	28.4 vs 6.4	0.23 (0.12-0.42)
≥30% vs <30%	50 vs 24	27.6 vs 10.3	0.36 (0.20-0.64)
≥40% vs <40%	39 vs 35	23.3 vs 14.5	0.72 (0.41-1.27)
**PFS**			
≥10% vs <10%	64 vs 10	8.1 vs 3.5	0.35 (0.17-0.73)
≥20% vs <20%	55 vs 19	8.2 vs 3.9	0.24 (0.13-0.43)
≥30% vs <30%	50 vs 24	8.2 vs 4.1	0.30 (0.17-0.52)
≥40% vs <40%	39 vs 35	8.2 vs 5.6	0.59 (0.35-1.01)

CI, confidence interval, *ETS* early tumor shrinkage, *HR* hazard ratio, *OS* overall survival, *pembrolizumab–chemotherapy* pembrolizumab plus chemotherapy, *PFS* progression-free survival

Online Resource 5 OS and PFS by DpR cutoffs in all randomized patients in the pembrolizumab–chemotherapy group

Categories	Patients, n	Median, months	HR (95% CI)
**OS**			
≥10% vs <10%	64 vs 10	21.0 vs 6.4	0.28 (0.13-0.59)
≥20% vs <20%	58 vs 16	27.6 vs 6.1	0.19 (0.10-0.36)
≥30% vs <30%	55 vs 19	28.4 vs 6.4	0.27 (0.15-0.49)
≥40% vs <40%	49 vs 25	28.4 vs 10.8	0.40 (0.22-0.70)
≥50% vs <50%	41 vs 33	28.5 vs 13.9	0.48 (0.27-0.84)
≥60% vs <60%	31 vs 43	35.8 vs 13.9	0.37 (0.20-0.68)
≥70% vs <70%	18 vs 56	NR vs 15.0	0.36 (0.16-0.80)
**PFS**			
≥10% vs <10%	64 vs 10	8.1 vs 3.5	0.35 (0.17-0.73)
≥20% vs <20%	58 vs 16	8.2 vs 3.0	0.23 (0.12-0.43)
≥30% vs <30%	55 vs 19	8.2 vs 3.9	0.22 (0.12-0.41)
≥40% vs <40%	49 vs 25	8.3 vs 4.0	0.27 (0.15-0.47)
≥50% vs <50%	41 vs 33	8.4 vs 4.2	0.31 (0.18-0.53)
≥60% vs <60%	31 vs 43	12.2 vs 4.3	0.24 (0.13-0.43)
≥70% vs <70%	18 vs 56	19.6 vs 6.0	0.23 (0.11-0.50)

CI confidence interval, *DpR* depth of response, *HR* hazard ratio, *NR* not reached, *OS* overall survival, *pembrolizumab–chemotherapy* pembrolizumab plus chemotherapy, *PFS* progression-free survival

Online Resource 6 Response by ETS and DpR cutoffs in all randomized patients in the pembrolizumab–chemotherapy group

Categories	Patients, n	Responders, n	DOR, median (range), months
**ETS**			
≥10% vs <10%	64 vs 10	42 vs 0	8.3 (1.2+ to 41.7+) vs NA
≥20% vs <20%	55 vs 19	42 vs 0	8.3 (1.2+ to 41.7+) vs NA
≥30% vs <30%	50 vs 24	40 vs 2	8.3 (1.2+ to 41.7+) vs 7.3 (4.2 to 10.5)
≥40% vs <40%	39 vs 35	30 vs 12	7.9 (1.2+ to 41.7+) vs 14.0 (3.2 to 33.2+)
**DpR**			
≥10% vs <10%	64 vs 10	42 vs 0	8.3 (1.2+ to 41.7+) vs NA
≥20% vs <20%	58 vs 16	42 vs 0	8.3 (1.2+ to 41.7+) vs NA
≥30% vs <30%	55 vs 19	42 vs 0	8.3 (1.2+ to 41.7+) vs NA
≥40% vs <40%	49 vs 25	39 vs 3	8.6 (1.2+ to 41.7+) vs 6.4 (3.2 to 10.5)
≥50% vs <50%	41 vs 33	35 vs 7	8.6 (1.2+ to 41.7+) vs 6.4 (3.2 to 23.0)
≥60% vs <60%	31 vs 43	29 vs 13	16.2 (1.2+ to 41.7+) vs 6.2 (2.3+ to 23.0)
≥70% vs <70%	18 vs 56	18 vs 24	17.5 (1.2+ to 41.7+) vs 6.2 (2.3+ to 33.2+)

DOR duration of response, *DpR* depth of response, *ETS* early tumor shrinkage, *NA* not applicable, *pembrolizumab–chemotherapy* pembrolizumab plus chemotherapy

Online Resource 7 Longitudinal percentage change from baseline in target lesion per RECIST v1.1 by investigator assessment in the pembrolizumab–chemotherapy group. (a) ETS cutoffs. (b) DpR cutoffs. For 3 patients who had only 1 imaging assessment before 63 days - 7 days from the randomization date, the first assessment was used. For 1 patient who had 2 imaging assessments after 63 days ± 7 days from the randomization date, the earlier assessment was used; both percentage decreases were less than 10%. *DpR* depth of response, *ETS* early tumor shrinkage, *pembrolizumab–chemotherapy* pembrolizumab plus chemotherapy, *RECIST v1.1* Response Evaluation Criteria in Solid Tumors version 1.1

(a)

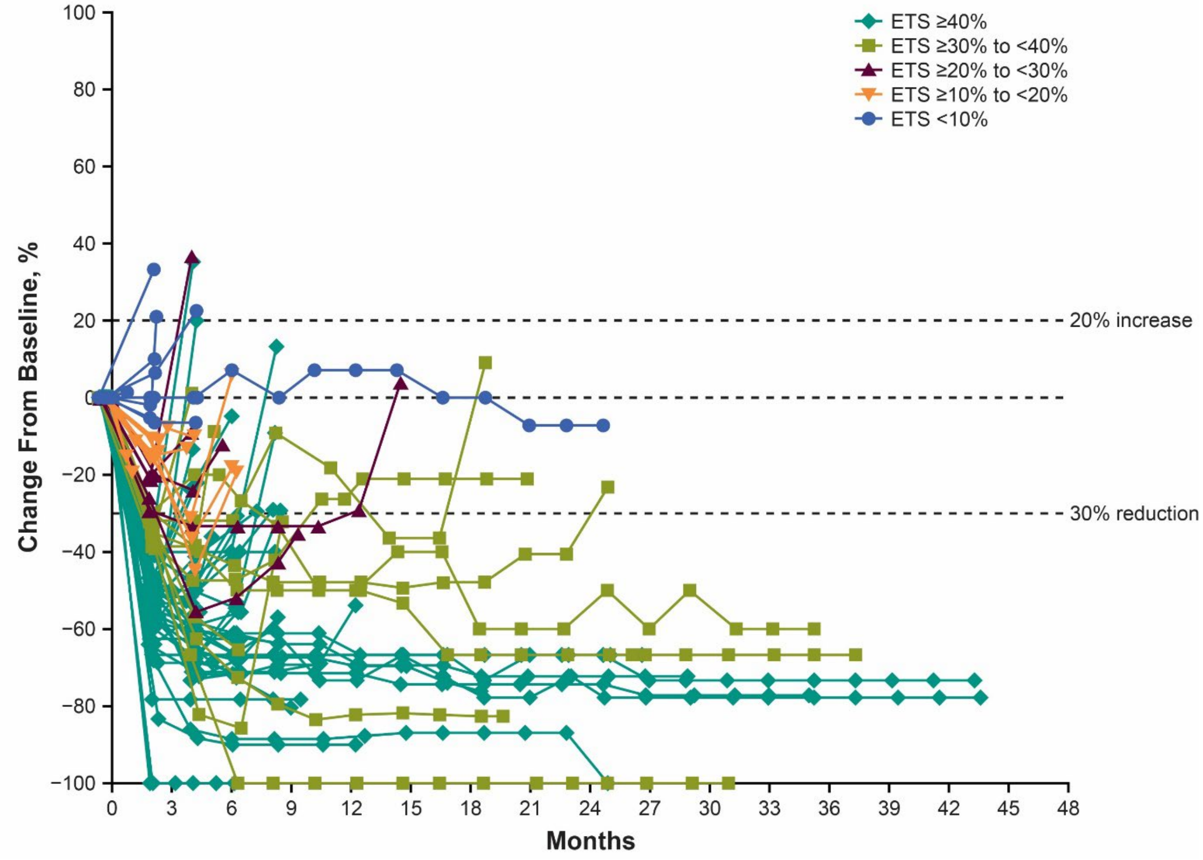

(b)

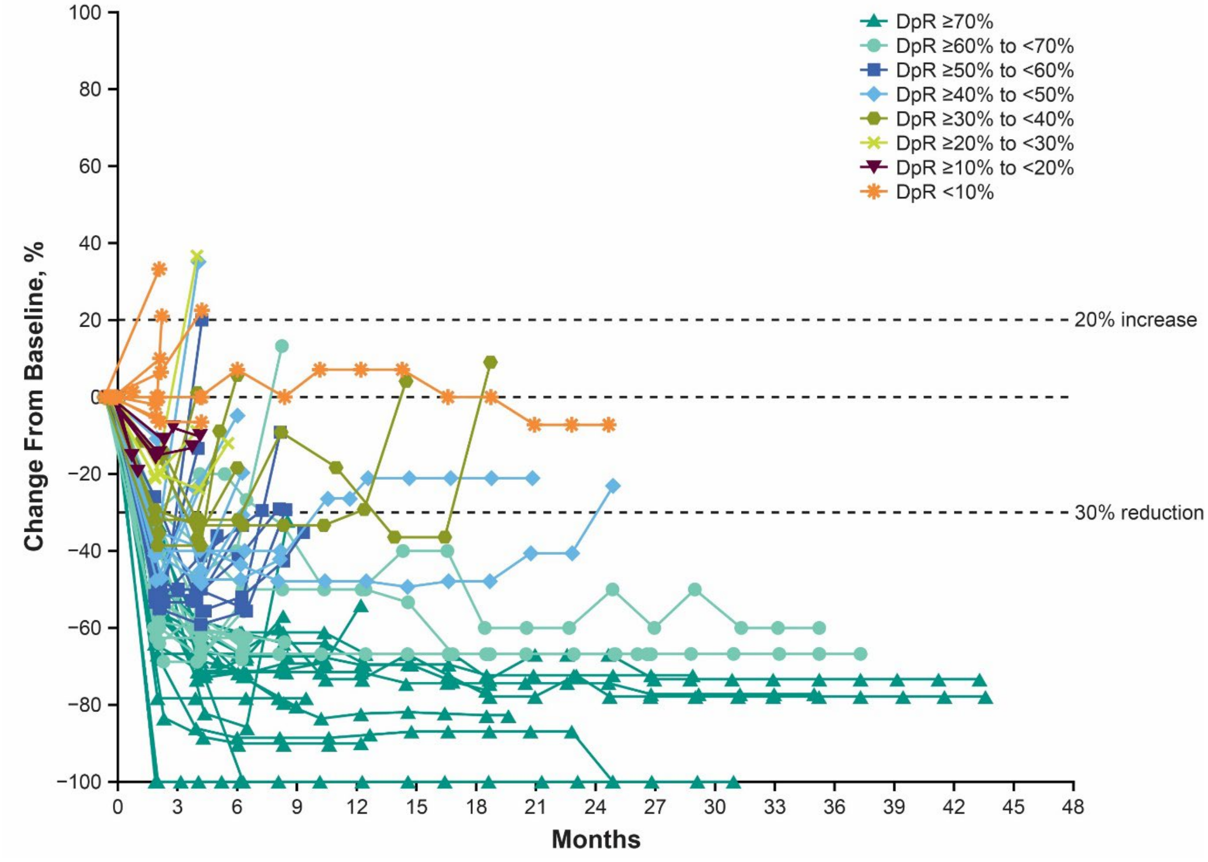

DpR ≥70%
DpR ≥60% to <70%
DpR ≥50% to <60%
DpR ≥40% to <50%
DpR ≥30% to <40%
DpR ≥20% to <30%
DpR ≥10% to <20%
DpR <10%
20% increase
30% reduction
Change From Baseline, %
100
80
60
40
20
0
−20
−40
−60
−80
−100
0 3 6 9 12 15 18 21 24 27 30 33 36 39 42 45 48
Months

Online Resource 8 Adverse event summary in Japanese patients

Events, n (%)	Pembrolizumab–chemotherapy<br>*n* = 74	Placebo–chemotherapy<br>*n* = 67
Any AEs	74 (100.0)	67 (100.0)
Grade 3-5	61 (82.4)	49 (73.1)
Led to discontinuation	20 (27.0)	15 (22.4)
Serious	32 (43.2)	32 (47.8)
Led to death	4 (5.4)	1 (1.5)
Treatment-related AEs[a]	73 (98.6)	66 (98.5)
Grade 3-5	55 (74.3)	41 (61.2)
Led to discontinuation	16 (21.6)	12 (17.9)
Serious	24 (32.4)	16 (23.9)
Led to death	2 (2.7)	1 (1.5)
Immune-mediated AEs and infusion reactions[b]	25 (33.8)	17 (25.4)
Grade 3-5	10 (13.5)	1 (1.5)
Led to discontinuation	7 (9.5)	2 (3.0)
Serious	12 (16.2)	1 (1.5)
Led to death	2 (2.7)	1 (1.5)

AE adverse event, *pembrolizumab–chemotherapy* pembrolizumab plus chemotherapy, *placebo–chemotherapy* placebo plus chemotherapy

[a]Determined by the investigator to be related to the drug.

[b]Immune-mediated AEs and infusion reactions were based on a list of preferred terms intended to capture known risks of pembrolizumab and were considered regardless of attribution to study treatment by the investigator.
